# Structure Analysis and Antioxidant Activity of a Novel Polysaccharide from Katan Seeds

**DOI:** 10.1155/2021/6349019

**Published:** 2021-01-12

**Authors:** Imen Trabelsi, Sirine Ben Slima, Naourez Ktari, Mohamed Bouaziz, Riadh Ben Salah

**Affiliations:** ^1^Laboratory of Microorganisms and Biomolecules (LMB), Centre of Biotechnology of Sfax, Road of Sidi Mansour Km 6, P.O. Box 1177, Sfax 3018, Tunisia; ^2^Laboratory of Enzyme Engineering and Microbiology, National School of Engineering of Sfax (ENIS), 1173-3038 Sfax, Tunisia; ^3^Department of Life Sciences, Faculty of Science of Gabes, Omar Ibn Khattab Street, Gabes 6029, Tunisia; ^4^Laboratoire d'Electrochimie et Environnement, Ecole Nationale d'Ingénieurs de Sfax, Université de Sfax, BP1173, 3038 Sfax, Tunisia

## Abstract

In the present work, a novel water-soluble polysaccharide (LWSP) was purified from Katan seeds. Polysaccharide was structurally characterized by NMR spectroscopic analysis, thin-layer chromatography (TLC), high-performance liquid chromatography (HPLC), Fourier-transform infrared spectroscopy (FTIR) analysis, X-ray diffraction (XRD), and UV absorption. TLC and HPLC showed that LWSP was a polysaccharide consisted mainly of glucose, mannose, xylose, and arabinose. The FTIR spectrum and UV absorption proved polysaccharide characteristic of LWSP. According to XRD, LWSP presented a semicrystalline behavior. The molecular weight was estimated as 64.56 kDa. Results obtained through ^13^C and ^1^H nuclear magnetic resonance (NMR) indicated that LWSP is consisted of four monosaccharide residues with *α* and *β* anomers. Physicochemical and antioxidant properties of LWSP were also investigated. Results revealed that LWSP exhibited interesting 1,1-diphenyl-2-picrylhydrazyl (DPPH) (IC_50_ = 4.48 mg/ml) and chelating activity (IC_50_ = 4.79 mg/ml), and it displayed moderate reductive capacities. Overall, the findings suggested that LWSP is a promising source of natural additives in various industries fields.

## 1. Introduction

Natural polymers as polysaccharides are generally obtained from marine organisms, fungal, bacterial, and vegetal sources. Plant polysaccharides may correspond to storage polymers or to molecules involved in the cell wall structure. Polysaccharides are a group of carbohydrates formed of monosaccharide units coupled by glycosidic linkage (*α* or *β* configuration). They are formed of high molecular-weight polymers [1]. They have found various applications in several industries due to their multifunctional bioactivities and physicochemical characteristics. Antioxidant molecules are able to inhibit the action of free radicals causing. These molecules are inadequate to prevent radical-induced damages [2]. In fact, some synthetic antioxidants such as butylated hydroxyl toluene (BHT) or butylated hydroxyl anisole (BHA) used in dietary supplements or in cosmetics have been suspected of being responsible for liver damage and carcinogenesis [2, 3]. The use of natural antioxidants extracted from plants in pharmaceutical and/or food application is more active than those obtained from chemical synthesis like BHT, BHA, or vitamin E.

Concerning pharmaceutical application, polysaccharides possess various biological activities including anticoagulant, anti-inflammatory, antitumor, antiviral, antipathogenic, and antioxidant activities and immune modulating [4]. These biological molecules presented many benefits for human health by reducing the risk of several diseases including heart disease, arthritis, and cancer [5]. Moreover, they are added in various food reformulations to obtain safety products with good quality [6]. In fact, they are used as natural preservatives in terms of antimicrobial and antioxidant agents, also as foaming and emulsifying ingredients, and they are added into diet foods due to their dietary fibers, mimetic fats, and prebiotic effects [7]. Many researchers have investigated the potential uses of polysaccharides extracted from vegetable processing waste such as potato starch waste [8], onion (*Allium cepa*) solid waste [9], and garlic (*Allium sativum* L.) [10] and from various plants such as fenugreek [11], chickpea [12], sorgho [6], and watermelon rinds [13].

Katan seeds are a member of the *Linaceae* family. The plant is native corps to West Asia and the Mediterranean. Katan is the seed obtained from the flax plant which named *Linum usitatissimum* L. [14]. Katan seeds presented high nutritional value due to dietary fibers, protein, alpha-linolenic fatty acids, and micronutrients [15]. In fact, chemical analysis of Katan seeds averaged 30–40% fat, 20–28% total dietary fibers, 20–25% protein, 3–4% ash, and 4–8% moisture and contain also vitamins such as A, B, D, and E, minerals, and amino acids [14]. Recently, many researchers have highlighted the essential roles of fiber viscosity as a factor that determines the gastrointestinal handling, essentially carbohydrate digestibility and absorption rates, which as a result impacts the glycemic response [15].

In this study, LWSP was purified and characterized by TLC, HPLC, RMN, X-ray diffraction, and UV visible. The physicochemical properties, the molecular weight, and antioxidant activities were also studied.

## 2. Materials and Methods

### 2.1. Material and Reagents

Katan seeds used in this study were purchased from the local market at Sfax city in Tunisia. Seeds were crushed in a Moulinex blender LM 241. The obtained powder was stored in clean and hermetic glass until use.

### 2.2. Extraction of Water-Soluble Polysaccharide (LWSP)

LWSP was extracted by the hot water technique as described by Liu et al. [16] with some modifications. Briefly, Katan seed powder was preextracted with 95% ethanol at room temperature to eliminate small molecules and impurities. The dry residue was extracted twice with 20 volumes of deionized water at 90°C while stirring for 4 h. The extract was combined and filtered, and filtrates were then evaporated under vacuum. The obtained liquid was precipitated with 95% (v/v) ethanol at 4°C for 24 h and then centrifuged (4500 × g) using a refrigerated centrifuge for 30 min (Hettich Zentrifugen, ROTINA 380R, Germany). Afterward, the precipitate was dried at 60°C for 3 h to obtain LWSP, and the polysaccharide yield (%, w/w) was calculated.

### 2.3. Physicochemical Characteristics

Various physicochemical properties like moisture, ash, fat, color, carbohydrate, protein, and pH (1% solution at 25 ± 0.5°C) of the extracted LWSP were determined.

The carbohydrate content was determined by phenol-sulfuric acid colorimetric method [17]. A standard curve was obtained using glucose standard (Sigma Aldrich, USA) at 5, 25, 50, 100, and 150 *μ*g/ml. The moisture, ash, and fat contents of LWSP were evaluated according to the AOAC methods [18]. Crude fat was determined gravimetrically after Soxhlet extraction of dried samples with hexane.

The pH of the 1% aqueous solution of extracted LWSP was measured by using a digital pH meter (Systronics Instruments, India) by immerging completely the glass electrode into the solution.

The sample CieLab parameters (*L*^∗^, *a*^∗^, and *b*^∗^) were read using a Color Flex spectrocolorimeter (Hunter Associates Laboratory Inc., Reston, VA, USA) and reported as *L*^∗^, *a*^∗^, and *b*^∗^ values, in which *L*^∗^ is a measure of lightness, *a*^∗^ represents the chromatic scale from green to red, and *b*^∗^ represents the chromatic scale from blue to yellow.

The average molecular weight was evaluated using a high-pressure gel filtration chromatograph equipped with a refractive index detector using Zorbax PSM 300 column (6.2 × 250) as previously described [19]. The average molecular weight (Mw) of the LWSP was determined by the comparison of its retention time with the calibration curve using different dextran with known molecular weights.

### 2.4. Scanning Electron Microscopy

The surface micromorphology of LWSP was observed using a scanning electron microscope system (JSM-5400, JEOL, Japan) with an accelerating voltage of 10.0 kV under 50x, 95x, and 250x magnifications. The double-sided adhesive coated aluminum SEM stub was used to fix the samples. After being frozen under liquid nitrogen, it was fractured, mounted, and sputtered with gold using a sputter coater (JFC-1100, JEOL, Japan) for conductivity. The LWSP samples were then photographed with an angle of 90° to the surface [20].

### 2.5. Monosaccharide Composition of LWSP

#### 2.5.1. Thin-Layer Chromatography (TLC)

LWSP (2 mg) was hydrolyzed in 250 *μ*l trifluoroacetic acid (4 M) for 8 h at 100°C. The hydrolysis LWSP was analyzed by TLC. The developing solvent was a mixture of chloroform/acetic acid/water (6 : 7 : 1). The revelation is obtained by spraying 5% (v/v) H_2_SO_4_ in ethanol followed by incubation and drying in oven at 105°C for 10 min. The standers used were as follows: glucose, fructose, sucrose, mannose, arabinose, and galactose at a concentration of 10 g/l.

#### 2.5.2. High-Performance Liquid Chromatography (HPLC)

The monosaccharide compositions were analyzed by HPLC using an Aminex HPX-87H column with a mobile phase of 0.001 N H_2_SO_4_. The sample was hydrolyzed by dissolved 2 mg of LWSP in 250 *μ*l of 4 M trifluoroacetic acid (TFA) at 100°C for 8 h. Then, 20 *μ*l of obtained hydrolysate was added to 980 *μ*l of H_2_O and filtered through a 0.45 *μ*m pore size filter. Monosaccharide composition was analyzed at a flow rate of 0.4 ml/min and at 60°C. Glucose, fructose, sucrose, gluconic acid, mannose, arabinose, galactose, and xylose were used as standard monosaccharide. The monosaccharide composition assays were performed in two independent experiments.

### 2.6. Structural Analysis of LWSP

#### 2.6.1. Fourier-Transformed Infrared Spectroscopy (FTIR) Analysis

The structure groups of the extracted LWSP were identified using Fourier-transformed infrared spectrophotometer (Nicolet FTIR spectrometer) equipped with a horizontal attenuated total reflection (ATR) accessory. 1 mg of dried sample was grounded with KBr powder and then pressed into 1 mm pellets for FTIR measurement from 4000 to 400 cm^−1^. The data were analyzed by the OPUS 3.0 data collection software program (Bruker, Ettlingen, Germany).

#### 2.6.2. Nuclear Magnetic Resonance (NMR) Analysis

The structural analysis of LWSP was carried out by ^1^H NMR and ^13^C NMR using a Bruker 600 M spectrometer (Rheinstetten, Germany) at 25°C. The 30 mg of powdered samples were dissolved in 1 ml 99.9% D_2_O. ^1^H NMR and ^13^C NMR spectra were recorded at a frequency of 300 and 75.5 MHz (field of 7.1 T), respectively. Data analysis was carried out using the MestRe Nova 5.3.0 (Mestrelab Research S.L.) software. The chemical shift was expressed in parts per million.

#### 2.6.3. X-Ray Diffraction

The physical characteristic of LWSP was studied to obtain the X-ray diffraction (XRD) pattern. It was employed using an X-ray diffractometer (Siemens D5000, Bruker, Germany). The data were collected in the 2 *θ* range 5–80° with a step size of 0.02° and a counting time of 0.78 s/step.

#### 2.6.4. UV Absorption Peak Detection

UV-visible spectra were determined using TU-1900 spectrophotometer at 25°C in the wavelength range of 200-800 nm [21]. The LWSP sample was dissolved in ultrapure water to a final concentration of 0.05%.

### 2.7. Antioxidant Activities of LWSP

#### 2.7.1. DPPH Radical-Scavenging Assay

The DPPH radical-scavenging activity of LWSP was determined according to the method described by Bersuder et al. [22]. Briefly, LWSP powder was dissolved in ultrapure water at different concentrations (0-10 mg/ml). 500 *μ*l of each sample was mixed with 375 *μ*l of 99.5% ethanol and 125 *μ*l of DPPH (0.02% in ethanol). Then, the mixtures were incubated in the dark at room temperature for 1 h, and the reduction of DPPH radical was measured in absorbance of 517 nm. BHT was used as a positive control. The DPPH radical-scavenging activity was calculated as follows:

(1) $$\begin{array}{c} \text{DPPH radical}‐\text{scavenging activity }\left(\%\right)=\frac{\left(A\text{control}-A\text{sample}\right)}{A\text{control}}*100, \end{array}$$

where *A*control is the absorbance of the control reaction and *A*sample is the absorbance of LWSP. All experiments were done in triplicate.

#### 2.7.2. Measurement of Reducing Power

The reducing power activity was determined by testing the reducing power of iron according to the method reported by Yildirim and Mavi [23]. Samples of LWSP were dissolved in 0.2 mol/l phosphate buffer (pH 6.6) at a series of concentrations (0-15 mg/ml). 2.5 ml of each sample was mixed with 2.5 ml of 10 mg/ml potassium ferricyanide, and the mixture was incubated at 50°C for 20 min. Then, 2.5 ml of 100 mg/ml trichloroacetic acid was added to the mixture. After centrifugation, 2.5 ml of the supernatant was mixed with 2.5 ml of distilled water and 0.5 ml of ferric chloride (0.1%, w/v). The mixture was incubated at room temperature for 10 min, and the absorbance was measured at 700 nm. Ascorbic acid was used as a positive control.

#### 2.7.3. Ferrous Ion Chelating Activity

The chelating of ferrous ions by LWSP was measured according to the previously reported method Decker and Welch [24]. Briefly, 3 ml of LWSP sample at different concentrations (0-10 mg/ml) was mixed with 100 *μ*l of FeCl_2_ (2 mM) and incubated at room temperature for 5 min. The reaction was initiated by the addition of 0.4 ml of 5 mM ferrozine solution. The mixture was shaken and incubated at room temperature for 10 min. The absorbance was measured at 562 nm using EDTA as a positive control. Analyses of all samples were run in triplicate and averaged. The chelating effect was calculated using the following equation:

(2) $$\begin{array}{c} \text{Ferrous ion}-\text{chelating activity }\left(\%\right)=\frac{\left(A\text{control}+A\text{blank}-A\text{sample}\right)}{A\text{control}}, \end{array}$$

where *A*control is the absorbance of the control (without LWSP), *A*blank is the absorbance of the LWSP (without ferrozine), and *A*sample is the absorbance of the mixture of LWSP and ferrozine.

## 3. Results and Discussion

### 3.1. Physicochemical Analysis of LWSP

The chemical composition of the LWSP is presented in Table 1. The pH of 1% LWSP solution recorded 7.00 ± 0.01. In addition, the results proved that carbohydrates presented the most interesting part (76.03 ± 0.06%) of the extract. The same result was obtained for the *sorghum* polysaccharides and *Opuntia Ficus Indica* cladode polysaccharides which recorded 78.84% and 85.31%, respectively [6, 25]. The ash and fat contents calculated for LWSP were 7.61 and 0.3%, respectively (Table 1). The sample was characterized by a relatively low moisture (3.83%).

The molecular weight of LWSP was investigated by the gel filtration high-performance liquid chromatography. LWSP samples showed two peaks which the major was determined to be approximately 64.56 kDa (Table 1). The presence of the minor peaks could be caused by the degradation of the molecules by the high temperature used for the dissolution of LWSP [25].

As presented in Table 1, LWSP showed a high value of *L*^∗^ 66.23. The *b*^∗^ and *a*^∗^ values were recorded at 14.56 and 0.53, respectively (Table 1). Similar results were obtained by Ktari et al. [11] who reported that polysaccharide extracted from fenugreek displayed the lighting yellow color. The incorporation of polysaccharides in food usually has an effect on the color of the final product [11]. This characteristic enhanced their suitability to be added in food and nonfood formulations.

### 3.2. Scanning Electron Microscopy

**Figure 1:**
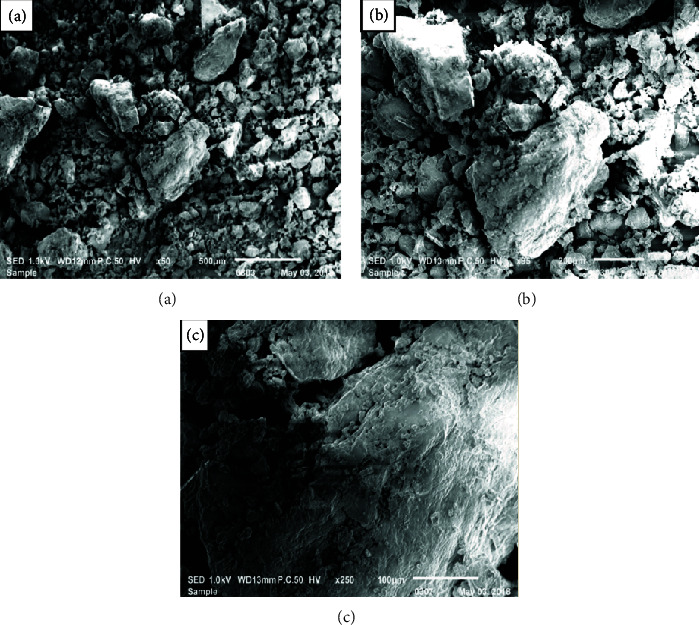
Scanning electron micrographs of the LWSP at magnifications of (a) 50x, (b) 95x, and (c) 250x, respectively.

The scanning electron microscopy (SEM) has been widely used to assess the surface morphology and complex 3D microstructure of polysaccharides for its application in different products. It was employed to monitor the morphological properties of the extracted polysaccharides. It is the most powerful tool in the study in structural morphology such as porosity, size, and shape of macromolecules [25–27]. SEM analysis of polysaccharide LWSP is presented in Figure 1. It is revealed as a sponge-like structure containing numerous cavities. Such structure makes the LWSP suitable for a variety of industrial applications. Also, this structure with cavity distribution allows the LWSP to absorb a large amount of water, when solubilized in water-based solutions. Such characteristic makes it a fast swelling system in several applications like gelling and emulsifying agents.

Previous works reported that the presence of numerous cavities on the surface of polysaccharides leads to an improvement in the various physical and functional characteristics, such as solubility, water/oil retention capacities, and emulsion properties. These characteristics are required for the application of such polysaccharides in various applications specifically in the food sector [27, 28]. In the other works, the scanning electron micrographs of gum polysaccharide extracted from Katan seeds revealed a splendor and shiny surface. In addition, the structure and surface morphology of polysaccharides could be influenced by different preparation methods: extraction and purification [29].

### 3.3. Monosaccharide Composition

**Figure 2:**
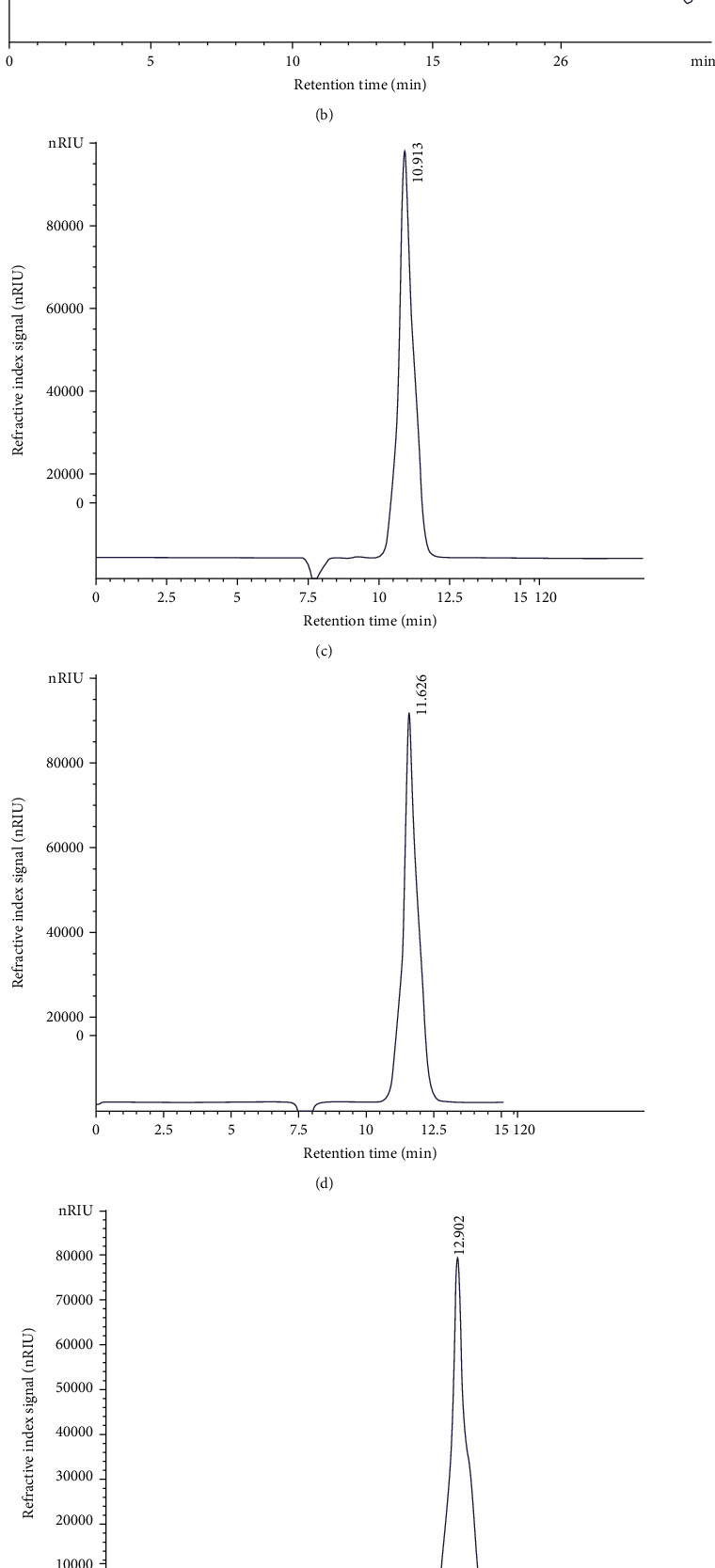
(a) TLC analysis of LWSP. (1) Arabinose, (2) xylose, (3) fructose, (4) glucose, (5) tagatose, (6) mannose, (7) rhamnose, (8) galactose, and (9) hydrolyzed LWSP. (b) HPLC analysis of LWSP hydrolyzed by TFA; (c) HPLC analysis of glucose; (d) HPLC analysis of mannose; (e) HPLC analysis of arabinose; (f) HPLC analysis of xylose.

The analysis of the hydrolysis LWSP obtained by TLC showed the presence of four plugs emerged with a retention factor of 0.55, 0.60, 0.63, and 0.69, respectively, similar to the standard monosaccharide glucose, mannose, arabinose, and xylose (Figure 2(a)). The obtained results showed that LWSP was a heteropolysaccharide, composed of glucose, mannose, xylose, and arabinose.

The monosaccharide composition of LWSP was also evaluated by comparing the retention time against standards using HPLC. The obtained chromatograms were presented in Figures 2(b)–2(e). It was revealed that LWSP was a polysaccharide composed of glucose, mannose, arabinose, and xylose. Mannose is the predominant peak with a retention time of 11.5 min followed by glucose (10.85 min). Arabinose and xylose are the minor components of LWSP. Accordingly, HPLC analysis confirms TLC.

In a previous study, it was reported that polysaccharide extracted from mature and ripe Katan seeds is composed of D-xylose, D-galacturonic acid, L-galactose, L-arabinose, L-rhamnose, and conceivably, some traces of D-glucose [30].

### 3.4. Structural Analysis of LWSP

#### 3.4.1. FTIR Spectroscopy

**Figure 3:**
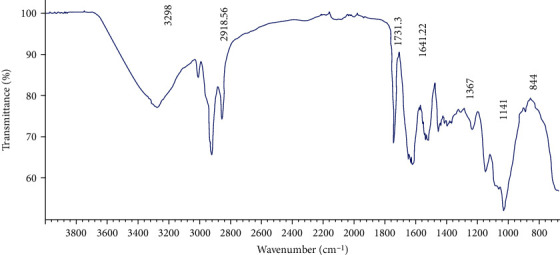
FTIR spectra of LWSP determined according to wavenumber (cm^−1^) and transmittance (%).

The FTIR spectrum was shown in Figure 3, a strong peak around 3298 cm^−1^ for hydroxyl groups stretching vibrations due to inter- and intramolecular hydrogen bands [31]. The peak around 2918.56 cm^−1^ showed an absorption for C-H stretching vibrations of the free sugars; the band around 1731.33 cm^−1^ showed an absorption for carboxyl groups stretching vibrations [32]; a peak around 1641.22 cm^−1^ was occurred due to the associated water [33]. Weak absorption peaks between 800 and 1200 cm^−1^ attributed to the presence of carbohydrate fingerprints and the identification of functional groups characterizing polysaccharides as stretching (C-O-C), bending (O-H), and deforming (CH_3_) vibrations [34]. The bands below 1000 cm also reported the visible bands' presence and/or possible linkages between molecules of monosaccharide [35]. In fact, the observed peak approximately at 844 cm^−1^ was attributed to be a characteristic of *α*-configuration in this polysaccharide [36].

#### 3.4.2. NMR Spectroscopy Data of LWSP

**Figure 4:**
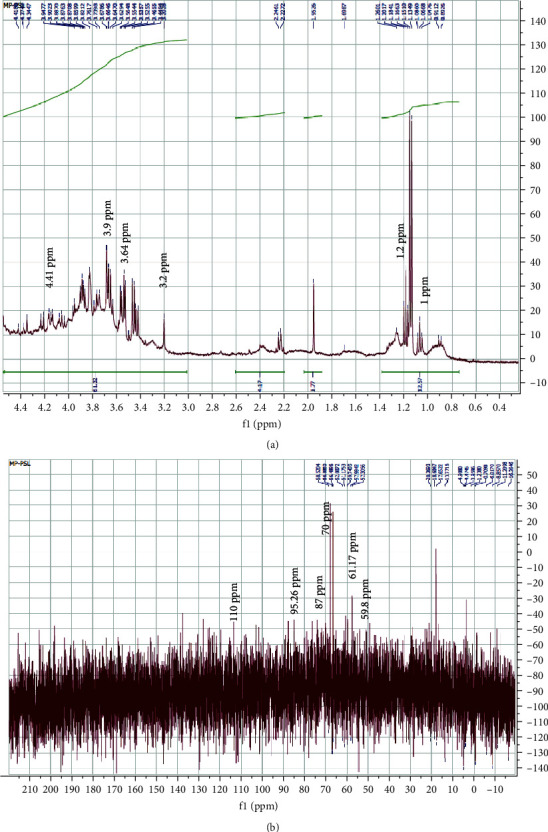
NMR spectrum of LWSP (a) ^13^C NMR spectrum and (b) ^1^H NMR spectrum. ^1^H NMR and ^13^C NMR spectra were recorded at a frequency of 300 and 75.5 MHz.

The structure of polysaccharide LWSP was elucidated through ^1^H and ^13^C NMR spectra (Figure 4). In ^1^H NMR spectrogram (Figure 4(a)), it showed a cramped region ranging between 3.2 and 4.41 ppm, indicating the presence of many similar sugar residues which confirm the presence of polysaccharides [37, 38]. Four proton resonance signal peaks can be observed in the anomeric proton region at 4.3, 3.9, 3.7, and 3.2 ppm, indicating that LWSP is consisted of four monosaccharide residues with *α* and *β* anomers. In fact, it was generally believed that the chemical shift values of *α*-anomeric protons were mostly larger than 4.0 ppm while the signals less than 4.0 ppm correspond to *β*-anomeric proton [39]. In previous data, it was reported that signals between 3.2 and 3.9 ppm could be attributed to the characteristics of H2-H5 resonate. Therefore, the absorption signal between 3.64 and 3.94 ppm was provoked by protons on sugar rings [40]. Intense peak signal observed at 1.0 and 1.2 ppm and identified the carbon group (R-CH2-CH3).

The ^13^C NMR spectrum (Figure 4(b)) showed the presence of sugar rings in our polysaccharides. In fact in this spectrogram, we found the existence of two anomeric carbons *α* and *β*-configurations in the regions of 95.26 ppm and 110.45 ppm, respectively. This finding confirms the results of ^1^H NMR spectrum. The absorption signals at 59.74 and 61.17 ppm found in the ^13^C NMR spectrum can be assigned to an O–CH3 group [12]. Also, we noticed that the presence of signal in the region ranging from 57 to 87 ppm can be assigned to sugars C2–C6 [41]. The signal appeared between 69 and 77.8 corresponding to the osidic groups (C2–C5) [42]. The strong signal positioned in the region of 60–80 ppm was qualified to the pyranose configuration in LWSP.

#### 3.4.3. X-Ray Diffraction Analysis of LWSP

XRD technique is usually used for semiquantitative and qualitative assessment of amorphous and semicrystalline and crystalline component. Indeed, the crystalline or noncrystalline characteristics of a substance play a major role in the physicochemical properties by influencing the structural arrangements, like solubility, viscosity, and flexibility.

**Figure 5:**
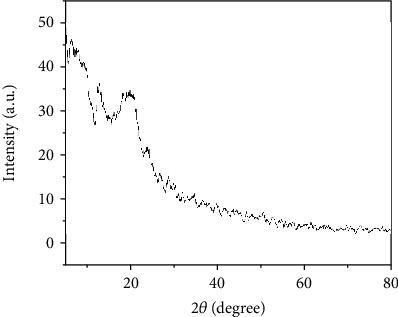
X-ray diffraction pattern of LWSP determined according to degree (2 *θ*) and intensity (a.u).

The X-ray diffractogram of LWSP was presented in Figure 5. Various sharp peaks ranging from 0 to 80° spectrum of 2 *θ* value were observed demonstrating the semicrystalline nature of LWSP. According to the literature, this finding was similar to the results obtained by Ben Slima et al. [6], and these authors noticed that water-soluble polysaccharides extracted from *Sorghum bicolor* seeds were semicrystalline fibers. In the other study reported by Rashid et al. [43], it showed that gum extracted *from* Katan seeds depicted amorphous behavior [44].

#### 3.4.4. UV-vis Spectroscopy

**Figure 6:**
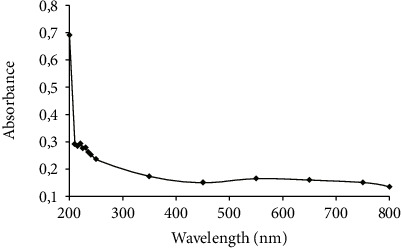
The absorbance of the purified LWSP within the wavelength range of 200-800 nm.

As presented in Figure 6, LWSP showed a maximum absorption peaks at 210 nm (Figure 6) and did not show any absorbance beyond that range. Thus, LWSP was identified as polysaccharides [45].

### 3.5. Antioxidant Activities of LWSP

#### 3.5.1. DPPH Radical-Scavenging Assay

**Figure 7:**
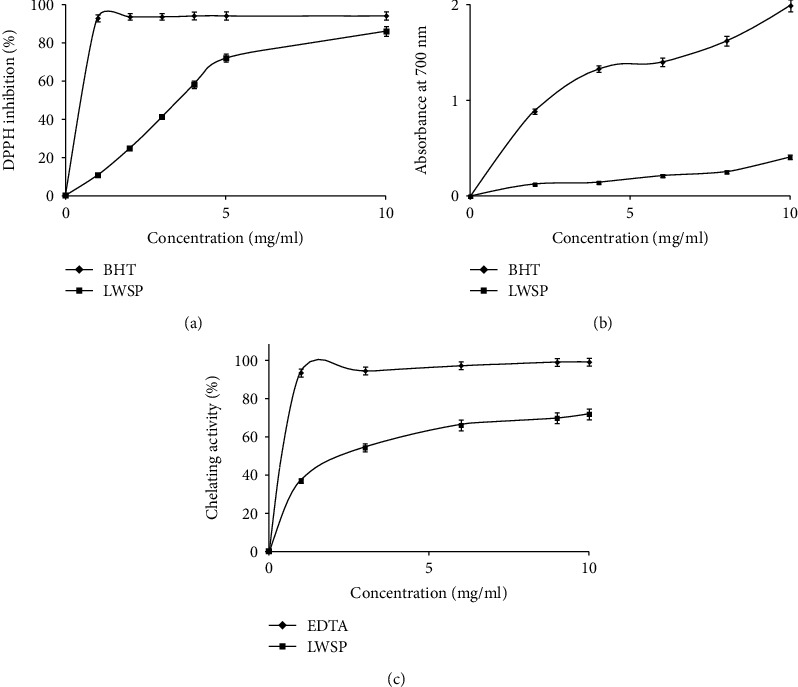
Antioxidant activities of LWSP: (a) the percentage of DPPH inhibition; (b) reducing power determined at A 700 nm; (c) the percentage of ferrous chelating activity made at different concentrations (0-10 mg/ml).

DPPH is a stable free radical used for screening the antioxidant ability of samples. The test mechanism is based on the reduction of DPPH by a proton-donating substrate [46]. The DPPH radical-scavenging capacity of LWSP and BHT (positive control) at different concentrations was determined as shown in Figure 7(a). LWSP activities were lower than that of BHT at the same concentration. The DPPH radical-scavenging activity of the LWSP increased as LWSP concentrations increased, and the highest DPPH radical-scavenging activity was (86.12%) obtained at 10 mg/ml. Our results were similar to those previously reported for other polysaccharides extracted from plants as the sorghum polysaccharides and [6] fenugreek polysaccharides [11]. The results implied that LWSP exhibits good antioxidant activities by transferring hydrogen atoms or electron to neutralize the free radicals [45]. The radical-scavenging activity of polysaccharides was depended on the molecular weight and monosaccharide composition [47]. In fact, the LWSP radical-scavenging ability could be attributed to the hydroxyl groups and carboxyl group existences in polysaccharides, which can act as quenching of the free radical [48].

#### 3.5.2. Reducing Power

The reducing capacity of sample is depended on the reductones' presence. The latter are capable to donate electrons to free radicals, break these oxidizing chain reactions, and prevent peroxide formation in order to make samples more stable [42].

The capacity of LWSP to reduce the oxidation form of iron (Fe^3+^) in ferric chloride to the ferrous form (Fe^2+^) by antioxidant was investigated. As demonstrated in Figure 7(b), the reducing power of LWSP appeared to be concentration dependent and reached a maximum of 0.408 at 10 mg/ml. Furthermore, LWSP showed lower reducing power than BHT. Our results were similar to those previously reported by Ben Slima et al. [42]. However, it was reported that polysaccharides extracted from *Hohenbuehelia serotina* exhibited reducing power about 0.50 at 10 mg/ml. It was reported that the differences in the monosaccharide components, chemical composition, molecular weight, glycoside bond types, and configuration of polysaccharide could affect its bioactivity [49].

#### 3.5.3. Ferrous Chelating Activity

Ferrous chelating assay is one of the essential antioxidant tests that provide significant reflection on the antioxidant activity. The chelating activity of LWSP was lower than EDTA which is a metal ion cheater (used as a positive control) at all concentrations (Figure 7(c)). At a concentration at 1 mg/ml, EDTA could chelate 94%, while the LWSP could chelate 37.6% of Fe ^2+^. In the activity of LWSP, a concentration-dependent response reaches a maximum of 72.31% at 10 mg/ml. The chelating activity of LWSP was higher than other plant polysaccharides such as polysaccharides from floral mushroom and *Cystoseira barbata* seaweed which recorded 42.68% at 5 mg/ml and 63% at of 10 mg/ml [30, 38]. It has been reported that the chelating activity is related to the large galactose and mannose quantities and the above functional groups presence in polysaccharide [11, 50].

## 4. Conclusion

The present study is aimed at identifying and characterizing novel polysaccharides extracted from Katan seeds by hot water technique as well as investigating their antioxidant activities. The obtained results from the spectroscopic analyses by SEM, FTIR, XRD, HPLC, and NMR showed that LWSP was a polysaccharide composed of xylose, arabinose, galactose, and glucoses with semicrystalline structure*. In vitro*, the reducing power, scavenging activity on DPPH, superoxide, and hydroxyl radical were also studied. Results demonstrated that LWSP exhibited higher antioxidant activity. These findings provided that the novel extracted polysaccharide might find promising values in functional products and therapeutic applications as an antioxidant agent.

## Figures and Tables

**Table 1:** Physicochemical analysis of LWSP.

Parameters	LWSP
Yield (%)	14.76 ± 0.99
pH	7.00 ± 0.01
Moisture (%)	3.83 ± 0.24
Ash (%)	7.61 ± 0.13
Fat (%)	0.3 ± 0.01
Carbohydrates (%)	76.03 ± 0.06
Mw	64.56 kDa
Color	
*a* ^∗^	0.53 ± 0.01
*b* ^∗^	14.56 ± 0.48
*L* ^∗^	66.23 ± 0.02

*L* ^∗^ is a measure of lightness, *a*^∗^ represents the chromatic scale from green to red, and *b*^∗^ represents the chromatic scale from blue to yellow.

## Data Availability

No data were used to support this study.

## References

[1] Trigui I., Yaich H., Sila A. (2018). Physicochemical properties of water-soluble polysaccharides from *black cumin* seeds. *International Journal of Biological Macromolecules*.

[2] Roy N., Laskar R. A., Ismail S., Kumar D., Ghosh T., Begum N. A. (2011). A detailed study on the antioxidant activity of the stem bark of Dalbergia sissoo Roxb., an Indian medicinal plant. *Food Chemistry*.

[3] Mengome L. E., Voxeur A., Akue J. P., Lerouge P. (2014). Screening of antioxidant activities of polysaccharides extracts from endemic plants in Gabon. *Bioactive Carbohydrates and Dietary Fiber*.

[4] Huang W., Deng H., Jin S., Ma X., Zha K., Xie M. (2018). The isolation, structural characterization and anti-osteosarcoma activity of a water soluble polysaccharide from _Agrimonia pilosa_. *Carbohydrate Polymers*.

[5] Andrade D., Gil C., Breitenfeld L., Domingues F., Duarte A. (2009). Bioactive extracts from *Cistus ladanifer* and *Arbutus unedo* L.. *Industrial Crops and Products*.

[6] Ben Slima S., Ktari N., Trabelsi I., Moussa H., Makni I., Ben Salah R. (2018). Purification, characterization and antioxidant properties of a novel polysaccharide extracted from *Sorghum bicolor* (L.) seeds in sausage. *International Journal of Biological Macromolecules*.

[7] Schmitt C., Kolodziejczky E. (2009). *Protein-Polysaccharide Complexes: From Basics to Food Applications Gums and Stabilisers for the Food Industry*.

[8] Rusendi D., Sheppard J. D. (1996). Hydrolysis of potato processing waste for the production of poly-*β*-hydroxybutyrate. *Bioresource Technology*.

[9] Kiassos E., Mylonaki S., Makris D. P., Kefalas P. (2009). Implementation of response surface methodology to optimise extraction of onion (*Allium cepa*) solid waste phenolics. *Innovative Food Science & Emerging Technologies*.

[10] Kallel F., Driss D., Chaari F. (2014). Garlic (*Allium sativum* L.) husk waste as a potential source of phenolic compounds: influence of extracting solvents on its antimicrobial and antioxidant properties. *Industrial Crops and Products*.

[11] Ktari N., Trabelsi I., Bardaa S. (2017). Antioxidant and hemolytic activities, and effects in rat cutaneous wound healing of a novel polysaccharide from fenugreek (*Trigonella foenum-graecum*) seeds. *International Journal of Biological Macromolecules*.

[12] Mokni Ghribi A., Sila A., Maklouf Gafsi I. (2015). Structural, functional, and ACE inhibitory properties of water-soluble polysaccharides from *chickpea flours*. *International Journal of Biological Macromolecules*.

[13] Ben Romdhane M., Haddar A., Ghazala I., Ben Jeddou K., Helbert C. B., Ellouz-Chaabouni S. (2017). Optimization of polysaccharides extraction from *watermelon* rinds: structure, functional and biological activities. *Food Chemistry*.

[14] Coşkuner Y., Karababa E. (2007). Some physical properties of flaxseed (*Linum usitatissimum* L.). *Journal of Food Engineering*.

[15] Vuksan V., Choleva L., Jovanovski E. (2017). Comparison of flax (*Linum usitatissimum*) and Salba-chia (*Salvia hispanica* L.) seeds on postprandial glycemia and satiety in healthy individuals: a randomized, controlled, crossover study. *European Journal of Clinical Nutrition*.

[16] Liu G., Xu S., Chen L. (2012). Chemical composition and bioactivities of a water-soluble polysaccharide from the endodermis of shaddock. *International Journal of Biological Macromolecules*.

[17] Hedge J. E., Hofreiter B. T., Whistler R. L., Miller J. N. B. (2011). *Carbohyd. chem*.

[18] AOAC (Association of Official Analytical Chemists) (2000). *Method of analysis Gaithersburg*.

[19] Bayar N., Kriaa M., Kammoun R. (2014). Extraction and characterization of three polysaccharides extracted from *Opuntia ficus indica* cladodes. *International Journal of Biological Macromolecules*.

[20] Maalej H., Moalla D., Boisset C. (2014). Rhelogical, dermal wound healing and in vitro antioxidant properties of exopolysaccharide hydrogel from *Pseudomonas stutzeri* AS22. *Colloids and Surfaces B: Biointerfaces*.

[21] He R., Zhao Y., Zhao R., Sun P. (2015). Antioxidant and antitumor activities *in vitro* of polysaccharides from *E. sipunculoides*. *International Journal of Biological Macromolecules*.

[22] Bersuder P., Hole M., Smith G. (1998). Antioxidants from a heated histidine-glucose model system. I: investigation of the antioxidant role of histidine and isolation of antioxidants by high performance liquid chromatography. *Journal of the American Oil Chemists' Society*.

[23] Yıldırım A., Mavi A., Bo B. (2001). Determination of antioxidant and antimicrobial activities of *Rumex crispus* L. extracts. *Journal of Agricultural and Food Chemistry*.

[24] Decker E. A., Welch B. (1990). Role of ferritin as a lipid oxidation catalyst in muscle food. *Journal of Agricultural and Food Chemistry*.

[25] Bayar N., Bouallegue T., Achour M., Kriaa M., Bougatef A., Kammoun R. (2017). Ultrasonic extraction of pectin from Opuntia ficus indica cladodes after mucilage removal: Optimization of experimental conditions and evaluation of chemical and functional properties. *Food Chemistry*.

[26] Alpizar-Reyes E., Carrillo-Navas H., Gallardo-Rivera R., Varela-Guerrero V., Alvarez-Ramirez J., Perez-Alonso C. (2017). Functional properties and physicochemical characteristics of tamarind (*Tamarindus indica* L.) seed mucilage powder as a novel hydrocolloid. *Journal of Food Engineering*.

[27] Kaushik P., Dowling K., Adhikari R., Barrow C. J., Adhikari B. (2017). Effect of extraction temperature on composition, structure and functional properties of flaxseed gum. *Food Chemistry*.

[28] Rashid F., Hussain S., Ahmed Z. (2018). Extraction purification and characterization of galactomannan from fenugreek for industrial utilization. *Carbohydrate Polymers*.

[29] Nep E. I., Conway B. R. (2011). Physicochemical characterization of grewia polysaccharide gum: effect of drying method. *Carbohydrate Polymers*.

[30] Nerkar P. P., Gattani S. (2010). *In vivo*, *in vitro* evaluation of linseed mucilage based buccal mucoadhesive microspheres of venlafaxine. *Drug Delivery*.

[31] Wu C. S. (2009). Renewable resource-based composites of recycled natural fibers and maleated polylactide bioplastic: characterization and biodegradability. *Polymer Degradation and Stability*.

[32] Wang Y. M., Wu F. J., Du L. (2014). Effects of polysaccharides from abalone (*Haliotis discus hannai Ino*) on HepG2 cell proliferation. *International Journal of Biological Macromolecules*.

[33] Park F. S. (1971). *Application of IR Spectroscopy in Biochemistry: Biology and Medicine*.

[34] Trabelsi L., M’sakni N. H., Ouada H. B., Bacha H., Roudesli S. (2009). Partial characterization of extracellular polysaccharides produced by cyanobacterium Arthrospira platensis. *Biotechnology and Bioprocess Engineering*.

[35] Parikh A., Madamwar D. (2006). Partial characterization of extracellular polysaccharides from cyanobacteria. *Bioresource Technology*.

[36] Ross K., Siow Y., Brown D., Isaak C., Fukumoto L., Godfrey D. (2014). Characterization of water extractable crude polysaccharides from cherry, raspberry, and ginseng berry fruits: chemical composition and bioactivity. *International Journal of Food Properties*.

[37] Khemakhem I., Abdelhedi O., Trigui I., Ayadi M. A., Bouaziz M. (2018). Structural, antioxidant and antibacterial activities of polysaccharides extracted from olive leaves. *International Journal of Biological Macromolecules*.

[38] Xu Y., Liu G., Yu Z. (2016). Purification, characterization and antiglycation activity of a novel polysaccharide from *black currant*. *Food Chemistry*.

[39] Zhou S. W., Liu H. L., Yan G., Xue H. Q., Guo W. (2016). NMR research of movable fluid and T2 531 cutoff of marine of South China. *Oil Gas Geology*.

[40] Sellimi S., Maalej H., Moalla Rekik D. (2018). Antioxidant, antibacterial and in vivo wound healing properties of laminaran purified from Cystoseira barbata seaweed. *International Journal of Biological Macromolecules*.

[41] Zhu J., Liu W., Yu J. (2013). Characterization and hypoglycemic effect of a polysaccharide extracted from the fruit of *Lyciumbarbarum* L. *Carbohydrate Polymers*.

[42] Ben Slima S., Trabelsi I., Ktari N. (2019). Novel Sorghum bicolor (L.) seed polysaccharide structure, hemolytic and antioxidant and laser burn wound healing effect. *International Journal of Biological Macromolecules*.

[43] Rashid F., Ahmed Z., Hussain S., Huang J.-Y., Ahmad A. (2019). *Linum usitatissimum L.* seeds: flax gum extraction, physicochemical and functional characterization. *Carbohydrate Polymers*.

[44] Hui H., Li X., Jin H., Yang X., Ruiming Zhao A. X., Qina B. (2019). Structural characterization, antioxidant and antibacterial activities of two heteropolysaccharides purified from the bulbs of *Lilium davidii var*.unicolor Cotton. *International Journal of Biological Macromolecules*.

[45] Williams P. A., Sayers C., Viebke C., Senan C., Mazoyer J., Boulenguer P. (2005). Elucidation of the emulsification properties of sugar beet pectin. *Journal of Agricultural and Food Chemistry*.

[46] Li X. L., Zhou A. G., Li X. M. (2007). Inhibition of *Lycium barbarum* polysaccharides and *Ganoderma lucidum* polysaccharides against oxidative injury induced by *γ*-irradiation in rat liver mitochondria. *Carbohydrate Polymers*.

[47] Feng S., Cheng H., Fu L. (2014). Ultrasonic-assisted extraction and antioxidant activities of polysaccharides from *Camellia oleifera* leaves. *International Journal of Biological Macromolecules*.

[48] Ronkart S. B., Blecker C. S., Deroanne C., Paquot M. (2009). Phénomène de la transition Vitreuse appliquée aux glucides alimentaires amorphes à l’état de poudre/Glass transition phenomena applied to powdered amorphous food carbohydrates. *Biotechnology, Agronomy, Society and Environment*.

[49] Parhat A. A., Paiheerding M., Yanhua G., Rano R., Haji A., Abulimiti Y. (2019). Sequential extraction, characterization and antioxidant activity of polysaccharides from Fritillaria pallidiflora Schrenk. *International Journal of Biological Macromolecules*.

[50] Thambiraj S. R., Phillips M., Koyyalamudi S. R., Reddy N. (2019). Yellow Lupin (*Lupinusluteus* L.) polysaccharides: antioxidant, immunomodulatory and prebiotic activities and their structural characterization. *International Journal of Food Properties*.

